# Supplementary material for: Implementing rapid, robust, cost-effective, patient-centred, routine genetic testing in ovarian cancer patients
Source: Sci Rep. 2016 Jul 13;6:29506. doi: 10.1038/srep29506 (PMC4942815; doi:10.1038/srep29506)
Supplement: Supplementary Information [file srep29506-s1.pdf]

Supplementary Information

Implementing rapid, robust, cost-effective, patient-centred, routine genetic testing in ovarian cancer patients

Angela George[1,2,3], Daniel Riddell[1], Sheila Seal[1,4], Sabrina Talukdar[1,4], Shazia Mahamdallie[1,4], Elise Ruark[1], Victoria Cloke[4], Ingrid Slade[5], Zoe Kemp[2], Martin Gore[3], Ann Strydom[1,4], Susana Banerjee[3], Helen Hanson[1,2] and Nazneen Rahman[1,2,4] for the Mainstreaming Cancer Genetics (MCG) programme

Supplementary Figures

Supplementary Figure 1. Example of Mainstream BRCA testing report.
The first page of the report is sent to the patient, the cancer team and the genetics team automatically. The second page, appendix is available on request and lists all the variants identified.

Supplementary Figure 2. Patient feedback questionnaires.

Supplementary Figure 3. *BRCA1* and *BRCA2* mutation testing guidelines used in Genetics.
In the standard BRCA testing pathway patients fulfilling the requirements below are eligible for referral to genetics and BRCA genetic testing. These are based on a threshold of 10% likelihood of having a BRCA mutation as determined using the Manchester scoring system. Further details and FAQs are available at www.icr.ac.uk/protocols

Supplementary Figure 4. Pedigrees of 33 ovarian cancer patients with BRCA mutations.
IDs correspond to those in Supplementary Table 1

Supplementary Figure 5. Management of BRCA carriers.
This is the standard protocol for management of BRCA carriers at RM Genetics unit. Further details and FAQs are available at www.icr.ac.uk/protocols

Supplementary Tables

Supplementary Table 1. 207 individuals offered BRCA testing through mainstream pathway.

Supplementary Table 2. Summary of patient feedback.

Supplementary Table 3. Summary of cancer team feedback.

Supplementary Appendix

Mainstream BRCA testing implementation kit – ovarian cancer

Supplementary Figure 1. Example of mainstream BRCA testing report

Report ID: 1XXX

TGL Clinical
Brookes Lawley Building
Institute of Cancer Research
15 Cotswold Road
Sutton, Surrey SM2 5NG

Email: tgl@icr.ac.uk
Phone: 020 8722 4122
Website: www.TGLclinical.com

CANCER PREDISPOSITION GENE TEST REPORT

Requesting clinician:	A. Doctor	Hospital ID:	65XXXX
Consultant:	A. Doctor	Patient NHS ID:	624 XXX XXXX
Department:	Gynaecology Unit	TGL sample ID:	15_000351_A
Hospital name:	RMH	Sample taken:	21/XX/20XX
Sample Type:	blood	Sample received:	22/XX/20XX 11:15

Referral reason: Ovarian cancer, age at diagnosis: 48

Test: BRCA1 and BRCA2 full gene analysis using the Illumina TruSight Cancer panel on blood-derived DNA

**Patient name:** **Maria** HASMUTATION	**DOB:**	**11/**XX**/19**XX

BRCA1 and BRCA2 Gene Test Results:

**BRCA1**	**c.1823delA**	**Pathogenic mutation detected**	**HET**
BRCA2	No pathogenic mutation detected		

Clinical implications:

• This provides a potential explanation for why this individual developed cancer.
• Review of result by clinician(s) to determine implications for clinical management of patient is recommended.
• Further information about the cancer risks is available at www.icr.ac.uk/protocols
• Review of patient by Genetics is recommended to discuss implications of this result for themselves and relatives.
• Testing for this mutation can be offered to relatives, through a Genetics department.

Reported: BC

Laboratory authorisation:

Clinical authorisation:

Notes:
Further information about methodologies and reporting procedures is available at www.TGLclinical.com
Methodology: Illumina TruSight cancer panel and Illumina sequencing, analysed by the GAMA and CIGMA bioinformatic pipelines. The full coding sequence and intron-exon boundaries are analysed for small variants and exon-level deletions and duplications. Pathogenic mutations and rare variants are confirmed by Sanger sequencing or Multiplex Ligation-dependant Probe Amplification (MLPA).
Reporting: Coding nomenclature based on the LRG reference sequences, where 1 is the A of the ATG translation initiation codon.
HET refers to a heterozygous mutation i.e. one that is present on only one allele.
A full list of variants identified in this individual is available as an Appendix to this report.

Page 1 of 1

Date issued: 21/XX/20XX

Further information is available at www.TGLclinical.com

Appendix to Report ID: 1XXX

TGL Clinical
Brookes Lawley Building
Institute of Cancer Research
15 Cotswold Road
Sutton, Surrey SM2 5NG

Email: tgl@icr.ac.uk
Phone: 020 8722 4122
Website: www.TGLclinical.com

CANCER PREDISPOSITION GENE TEST REPORT - APPENDIX

Full List of Variants

**Patient name:** **Maria** HASMUTATION	**DOB:** **11/**XX**/19**XX

Requesting clinician:	A. Doctor	Hospital ID:	6XXXX
Consultant:	A. Doctor	Patient NHS ID:	624 XXX XXXX
Department:	Gynaecology Unit	TGL sample ID:	15_000351_A
Hospital name:	RMH	Sample taken:	21/XX/20XX
Sample Type:	blood	Sample received:	22/XX/20XX 11:15

BRCA1

Pathogenic mutation: c.1823delA heterozygous

Other variants: No clinical action in this individual or predictive testing in their relatives is currently recommended for these variants. Gene variants are common in the normal population.

Gene	Variant	Zygosity
BRCA1	c.2311T>C_p.=	heterozygous
BRCA1	c.2612C>T_p.Pro871Leu	heterozygous
BRCA1	c.3119G>A_p.Ser1040Asn	heterozygous
BRCA1	c.4308T>C_p.=	heterozygous

BRCA2

No pathogenic mutation

Other variants: No clinical action in this individual or predictive testing in their relatives is currently recommended for these variants. Gene variants are common in the normal population.

Gene	Variant	Zygosity
BRCA2	c.1114A>C_p.Asn372His	heterozygous
BRCA2	c.4563A>G_p.=	homozygous
BRCA2	c.6513G>C_p.=	homozygous
BRCA2	c.7397T>C_p.Val2466Ala	homozygous

Appendix Page 1 of 1

Date issued: 21/XX/20XX

Further information is available at www.TGLclinical.com

Supplementary Figure 2. Patient feedback questionnaires.

I was given a Patient Information Sheet prior to my test.

The written information provided was clear and helpful.

I was clear in my own mind why I was being offered a genetic test.

I was given sufficient time to think about whether I wanted a test.

I was aware that I could have additional discussions with the Genetics Team before deciding whether to have a test.

I was aware that the result of the test might impact me.

I was aware that the result of the test might have implications for my family.

I was aware that further discussions with the Genetics Team would be organised for me if a gene mutation was found.

I was informed when the result would be available.

I was informed how I would receive the result.

Questionnaire v.1 no mutation

Questionnaire v.2 no mutation

- I was happy to receive my results by letter.
- I was happy to receive a copy of my test report (results).

(Questionnaire v.1 and v.2, no mutation)

- When I received my result I was given a Patient Information Sheet about receiving a normal BRCA1 and BRCA2 result.
- I understood the implications of this result for me.

Questionnaire v.1 mutation

- When I received my result, I was told I would be sent an appointment to see the Genetics Team.
- Within a month of receiving my result I had been sent a date for an appointment with the Genetics Team.

Questionnaire v.2 mutation

- I was happy to receive my results by letter.
- I was happy to receive a copy of my test report (results).
- When I received my result, I also received an appointment with the Genetics Team.
- When I received my result, I also received a Patient Information Sheet about receiving a BRCA1 and BRCA2 test result that identifies a mutation.

(Questionnaire v.1 and v.2, mutation)

- My appointment with the Genetics Team helped me to understand what the result meant for me and for my family.

(All questionnaires)

I was happy to have the genetic test at one of my existing Oncology appointments rather than in a separate appointment with the Genetics Team.

I am pleased I had the genetic test.

Do you have any other comments or suggestions for how we might improve the service?

Note: **See Supplementary Table 2 for questionnaire responses**

Supplementary Figure 3. *BRCA1* and *BRCA2* mutation testing guidleines used in Genetics.

Protocol 2
BRCA1 and *BRCA2* mutation testing guidelines

A
Woman with **breast cancer** who →

1) has bilateral BC and both cancers diagnosed < 50 yrs
2) has triple negative BC diagnosed < 50 yrs
3) has OC
4) has bilateral BC and a relative with BC < 60 yrs
5) has a relative with BC and both diagnosed < 45 yrs
6) has relatives with cancer and a Manchester Score ≥15

B
Woman with **ovarian cancer** who →

1) has BC
2) has a relative with OC or MBC
3) has relatives with cancer and a Manchester Score ≥ 15

C
Male with **breast cancer** who →

1) has a relative with OC or MBC
2) has relatives with cancer and a Manchester Score ≥ 15

D
Individual **unaffected** with cancer who →

1) meets unaffected testing criteria *

Results
***BRCA1/2* mutation identified:** see Protocol 3
***BRCA1/2* mutation not identified:** see Protocol 1 for breast screening recommendation
***BRCA1/2* variants/VUS identified:** see Protocol 4 and email: vus@icr.ac.uk

Notes*
The above criteria relate to a full gene screen of *BRCA1* and *BRCA2,* individuals of Ashkenazi Jewish heritage may be eligible for founder mutation testing.*

Key
Relative = first degree or second degree relative only, except when calculating a Manchester score*. Female relatives through an intervening male shift up one degree of relationship*
BC = breast cancer
MBC = male breast cancer
OC = ovarian cancer
Triple negative breast cancer = breast tumour negative for oestrogen receptor (ER), progesterone receptor (PR) and HER2 expression

Manchester Score*	
**Cancer, age at diagnosis**	**Score**
♀ Breast Cancer, <30	11
♀ Breast Cancer, 30-39	8
♀ Breast Cancer, 40-49	6
♀ Breast Cancer, 50-59	4
♀ Breast Cancer, > 59	2
♂ Breast Cancer, <60	13
♂ Breast Cancer, > 59	10
Ovarian Cancer, <60	13
Ovarian Cancer, >59	10
Pancreatic Cancer	1
Prostate Cancer, <60	2
Prostate Cancer, >59	1

** See FAQ document. http://www.icr.ac.uk/protocols*

01/01/2013

In the standard BRCA testing pathway patients fulfilling the requirements above are eligible for referral to genetics and BRCA testing. These are based on a threshold of 10% likelihood of having a BRCA mutation as determined using the Manchester scoring system. Futher details and FAQs are available at www.icr.ac.uk/protocols

Supplementary Figure 4. Pedigree of 33 ovarian cancer patients with BRCA mutations.

Key: = ovarian cancer   or   = breast cancer   or   = other cancer   = proband

ID_003

BRCA2_Exon 21-24 deletion

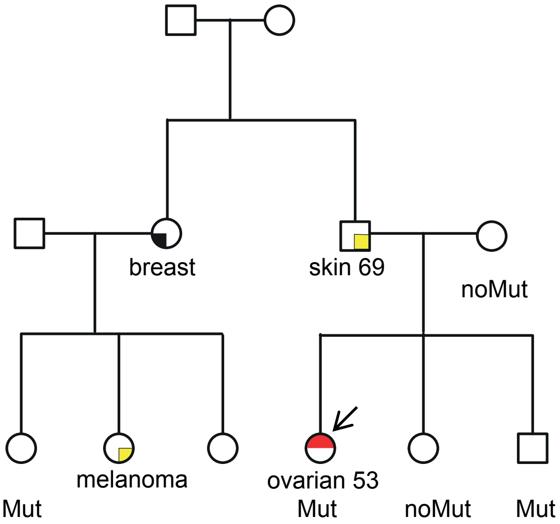

ID_009

BRCA2_c.3319C>T_p.Gln1107X

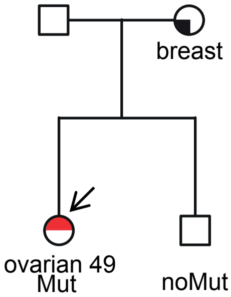

ID_028

BRCA2_c.5279C>G_p.Ser1760X

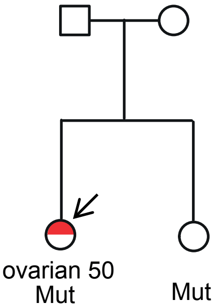

ID_014

BRCA1_c.3607C>T_p.Arg1203X

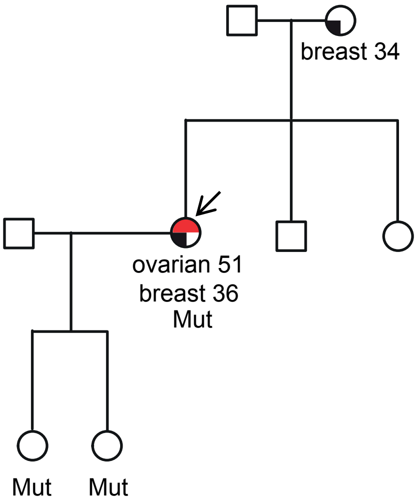

ID_036

BRCA2_c.4398_4402delACATT

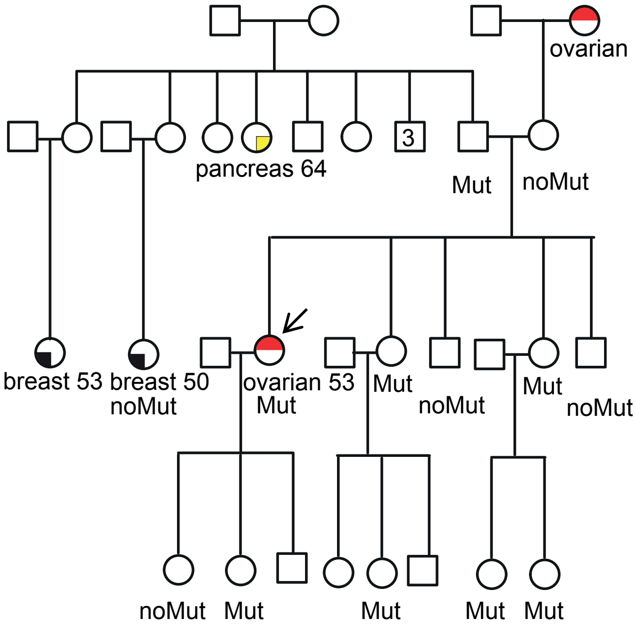

ID_034

BRCA1_c.5266dupC

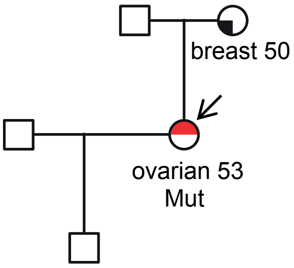

IDs correspond to those in Supplementary Table 1

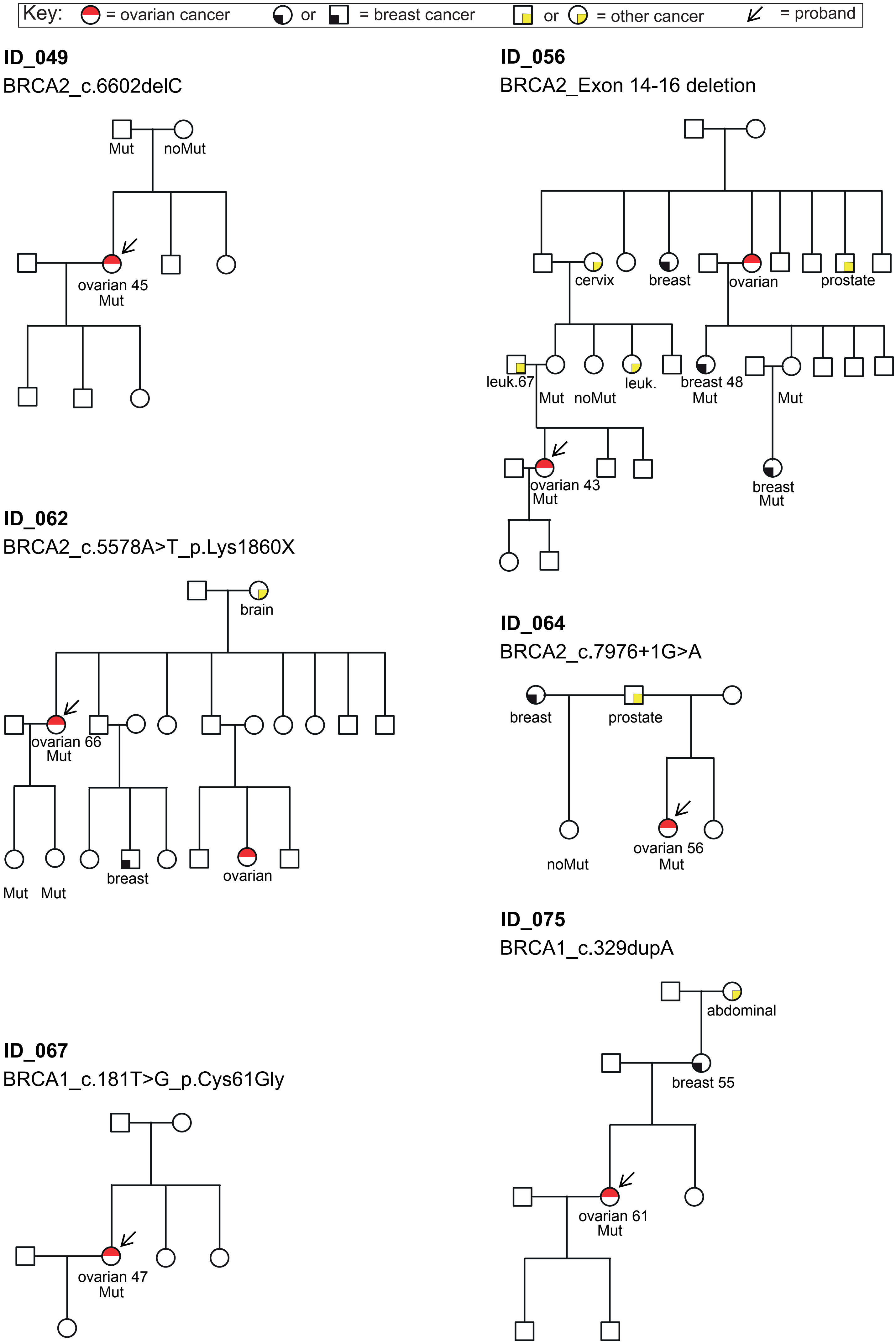
Key: = ovarian cancer or = breast cancer or = other cancer = proband
ID_049
BRCA2_c.6602delC
Mut
noMut
ovarian 45
Mut
ID_056
BRCA2_Exon 14-16 deletion
cervix
breast
ovarian
prostate
leuk.67
Mut
noMut
leuk.
breast 48
Mut
Mut
ovarian 43
Mut
breast
Mut
ID_062
BRCA2_c.5578A>T_p.Lys1860X
brain
ovarian 66
Mut
breast
ovarian
Mut
Mut
ID_064
BRCA2_c.7976+1G>A
breast
prostate
ovarian 56
Mut
noMut
ID_075
BRCA1_c.329dupA
abdominal
breast 55
ovarian 61
Mut
ID_067
BRCA1_c.181T>G_p.Cys61Gly
ovarian 47
Mut

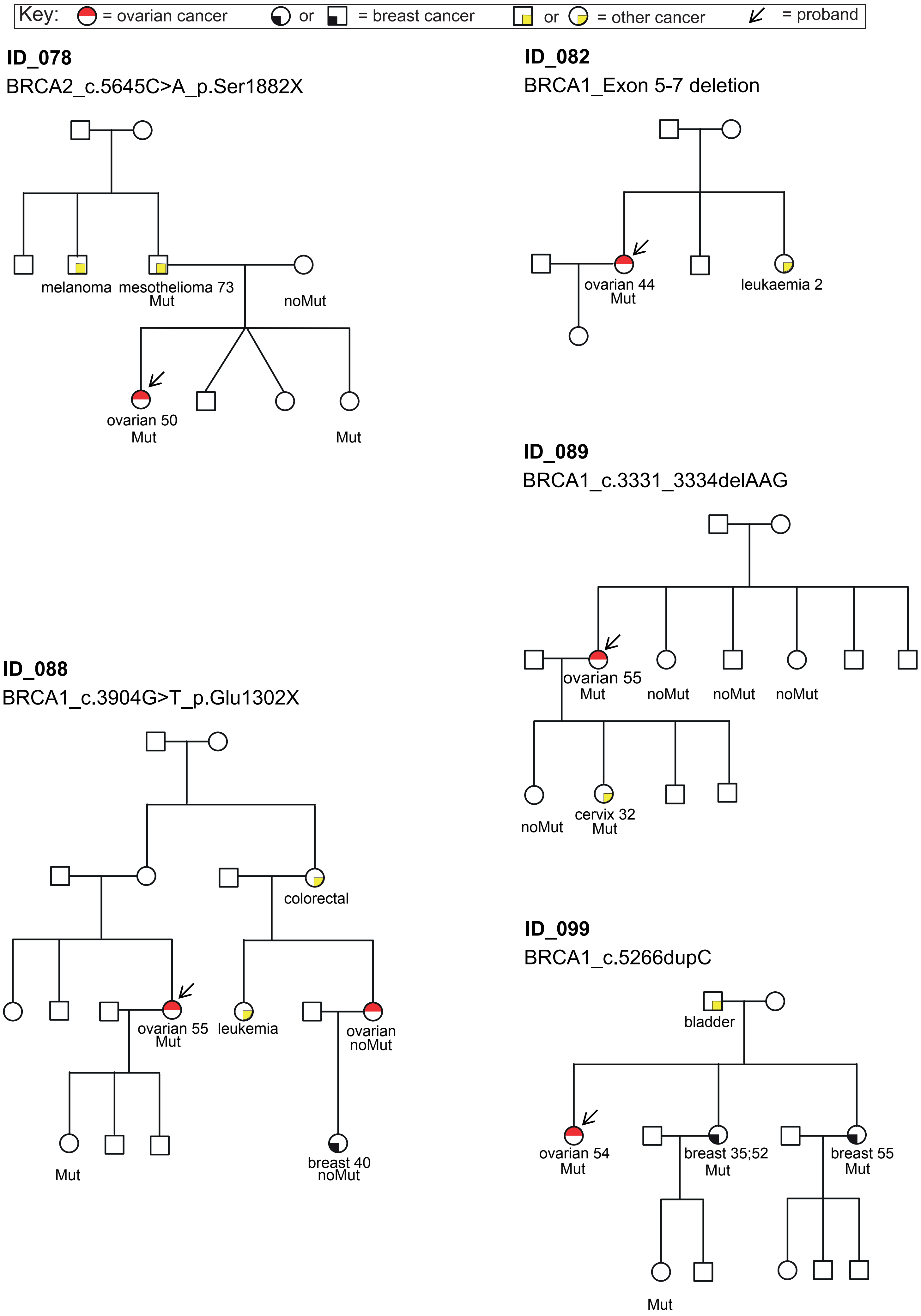

IDs correspond to those in Supplementary Table 1

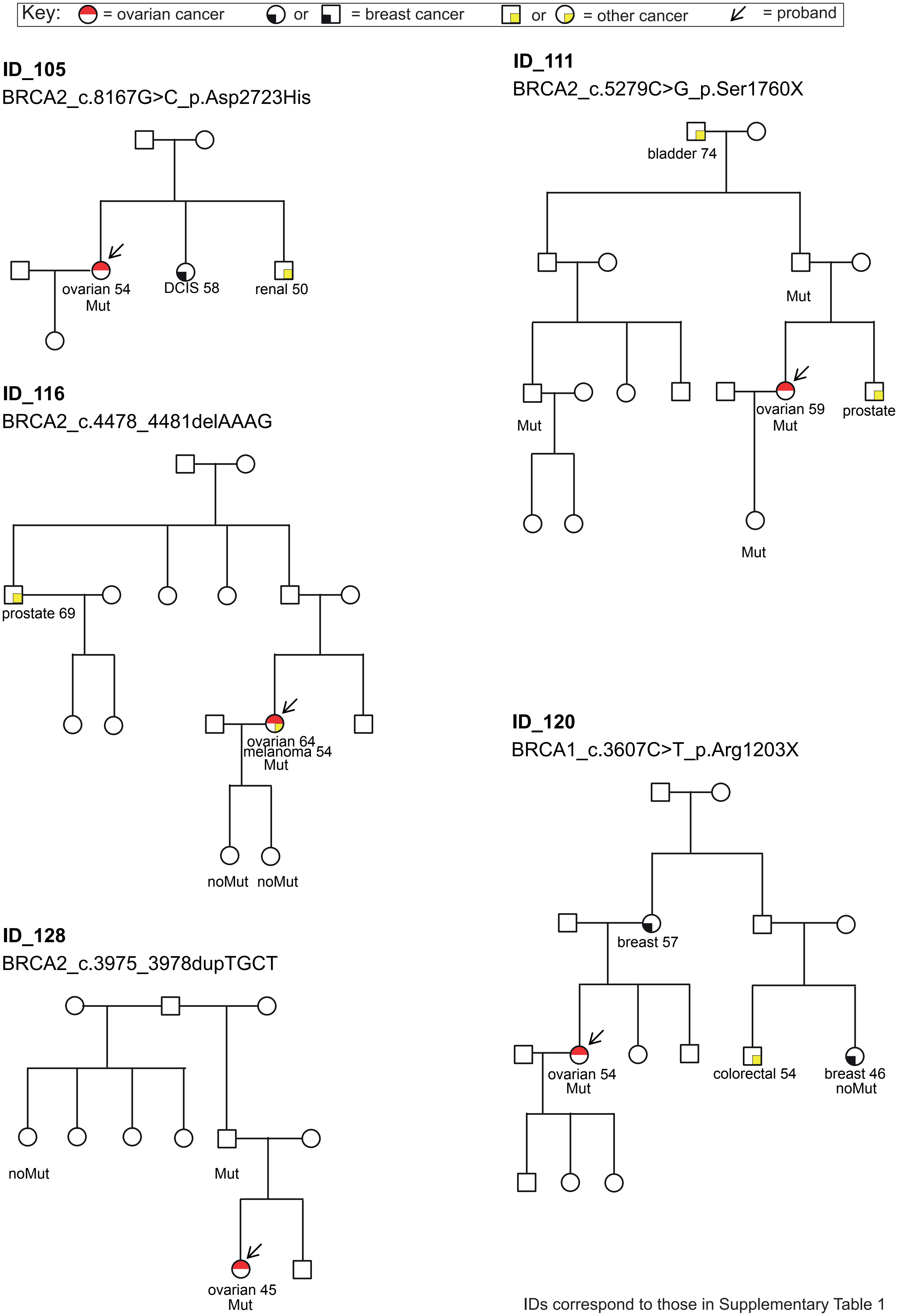

IDs correspond to those in Supplementary Table 1

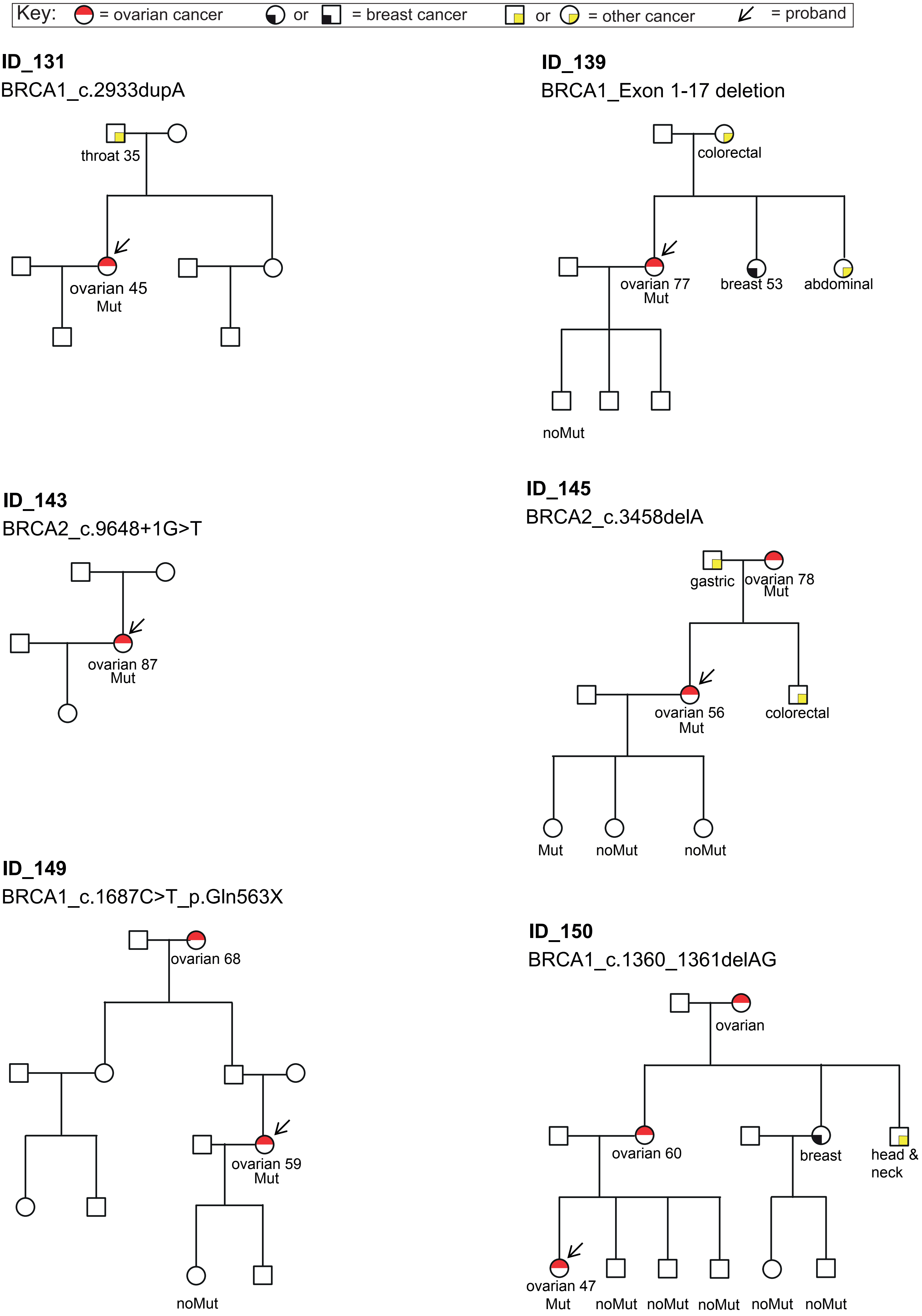
Key: = ovarian cancer or = breast cancer or = other cancer = proband
ID_131
BRCA1_c.2933dupA
throat 35
ovarian 45
Mut
ID_139
BRCA1_Exon 1-17 deletion
colorectal
ovarian 77
Mut
breast 53
abdominal
noMut
ID_143
BRCA2_c.9648+1G>T
ovarian 87
Mut
ID_145
BRCA2_c.3458delA
gastric
ovarian 78
Mut
ovarian 56
Mut
colorectal
Mut
noMut
noMut
ID_149
BRCA1_c.1687C>T_p.Gln563X
ovarian 68
ovarian 59
Mut
noMut
ID_150
BRCA1_c.1360_1361delAG
ovarian
ovarian 60
breast
head &
neck
ovarian 47
Mut
noMut
noMut
noMut
noMut
noMut

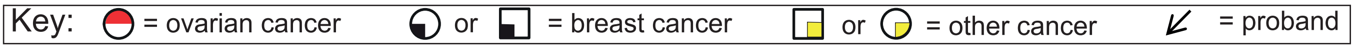

ID_156

BRCA1_c.5266dupC

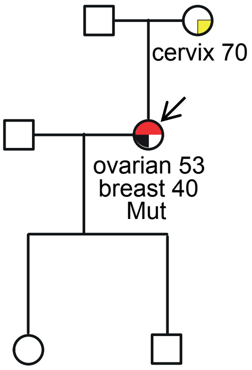

ID_163

BRCA1_c.4527C>G_p.Tyr1509X

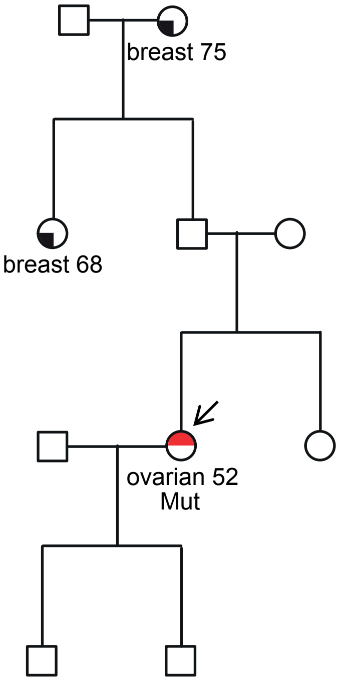

ID_182

BRCA1_c.5266dupC

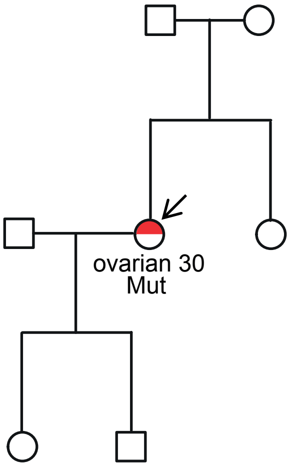

ID_202

BRCA1_c.2612_2613insT

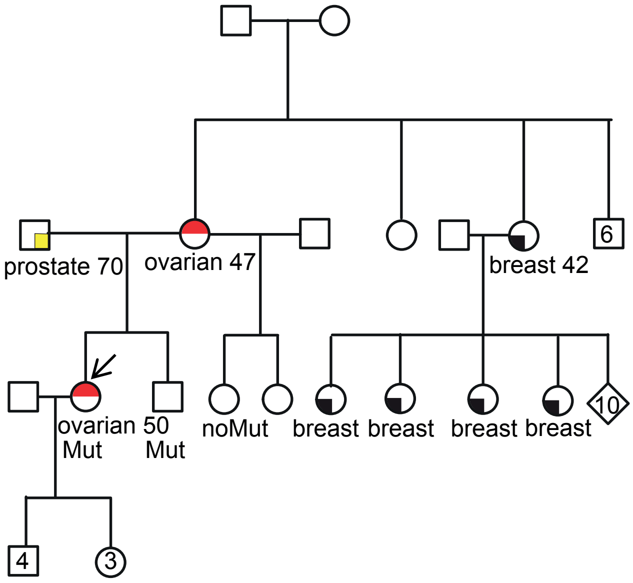

ID_205

BRCA2_c.2330dupA

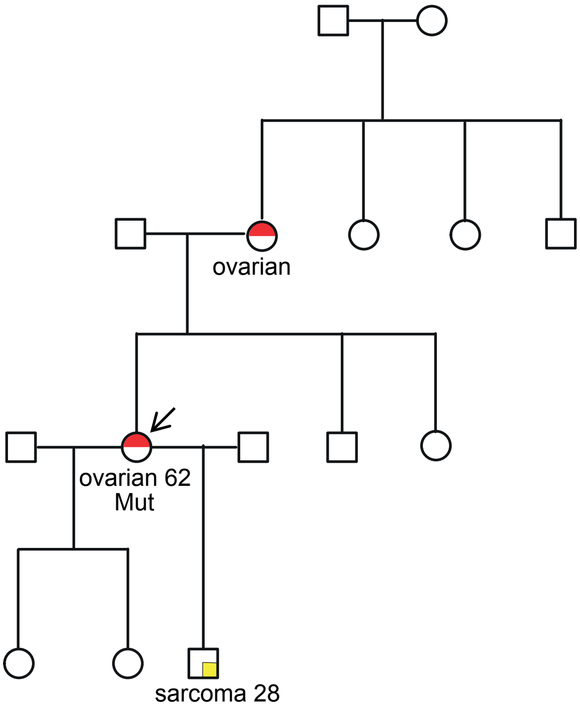

IDs correspond to those in Supplementary Table 1

Supplementary Figure 5. Management of BRCA carriers.

Protocol 3
BRCA mutation carrier guidelines

BRCA1 and/or *BRCA2* mutation analysis

Negative result
no mutation identified
Breast screening according to Protocol 1

VUS
variant of unclear significance identified
CIGMA report issued, see protocol 2 FAQ

Positive result
clearly pathogenic mutation identified

BRCA1 risks*

Unaffected carriers:

Breast Cancer
Lifetime risk (to 80 yrs): 60%-90%
Average remaining lifetime and 5 year risks - see table below

Ovarian cancer
Lifetime risk: 40%-60%
Majority of lifetime risk conferred after age 40

Male breast cancer
Lifetime risk ~0.1-1%

Prostate cancer
Lifetime risk similar to population risk ~8%

Affected carriers:

Women with unilateral breast cancer
Lifetime risk contralateral breast cancer ~50%
Overall 5 year risk ~10%

BRCA2 risks*

Unaffected carriers:

Breast Cancer
Lifetime risk (to 80 yrs): 45%-85%
Average remaining lifetime and 5 year risks - see table below

Ovarian cancer
Lifetime risk: 10%-30%
Majority of lifetime risk conferred after age 50

Male breast cancer
Lifetime risk ~5-10%

Prostate cancer
Lifetime risk ~25%

Affected carriers:

Women with unilateral breast cancer
Lifetime risk contralateral breast cancer ~50%
Overall 5 year risk ~5-10%

BRCA1*-Breast cancer risks

Current age (yrs)	Approximate remaining lifetime risk to 80 yrs	Approximate 5 yr risk
20-25	70%	5%
26-30	70%	5%
31-35	65%	5%
36-40	65%	10%
41-45	60%	10%
46-50	55%	15%
51-55	50%	15%
56-60	40%	10%
61-65	30%	10%
66-70	25%	10%

BRCA2*-Breast cancer risks

Current age (yrs)	Approximate remaining lifetime risk to 80 yrs	Approximate 5 yr risk
20-25	70%	~1%
26-30	70%	2%
31-35	65%	5%
36-40	65%	5%
41-45	60%	10%
46-50	55%	10%
51-55	50%	10%
56-60	50%	10%
61-65	45%	15%
66-70	35%	15%

Carrier management

Screening*

Breast
Advise on breast awareness
Annual mammography 40-70 yrs - review age 70
Annual MRI 30-50 years
No breast screening for male carriers

Ovary
Ovarian screening is not recommended

Other cancers
No screening is recommended for other cancers
Carriers may be eligible for screening in research studies

Chemoprevention*

- It is not appropriate to offer chemoprevention to *BRCA1* carriers.
- Discuss benefits and sides effects with *BRCA2* carriers. Can be offered if no contra-indications.

Risk-reducing surgery*

Breast
Discuss bilateral risk-reducing mastectomy
- breast cancer risk reduction 90-95%
- refer to surgeon to discuss surgical options

Ovary
Offer risk-reducing bilateral salpingo-oophorectomy, once childbearing is complete.
Discuss the following:
- Ovarian cancer risk reduction ~95%
- Breast cancer risk reduction ~ up to 50%
- Side effect: premature menopause
- HRT should be offered until age 50 in women who have not had ER positive breast cancer

* *See FAQ document. http://www.icr.ac.uk/protocols*

01/11/2013

This is the standard protocol for management of BRCA carriers at Royal Marsden Genetics Unit.
Further details and FAQs are available at www.icr.ac.uk/protcols

Supplementary Table 1. 207 individuals offered BRCA testing through mainstream pathway.

ID	Age at OC	Grade	Stage	Histology	Timing of testing	Other cancer	Family History of BC/OC	Mutation status	How test influenced management
001	56	high	3	serous	relapse		no	no mutation	trial
002	53	high	3	serous	remission (later relapsed)		no	no mutation	chemo choice
003	52	high	2	serous	remission		yes	BRCA2 deletion exon 21-24	breast surveillance
004	49	high	3	serous	relapse		no	no mutation	trial
005	61	high	3	serous	remission (later relapsed)		no	no mutation	trial
006	49	high	3	endometroid	relapse		no	no mutation	trial
007	57	high	4	serous	relapse		no	no mutation	trial
008	63	high	3	serous	relapse		no	no mutation	trial
009	47	high	3	serous	remission (later relapsed)		yes	BRCA2 c.3319C>T_p.Gln1107X	breast surveillance
010	59	high	2	serous	remission		no	no mutation	
011	64	high	3	serous	relapse		no	no mutation	trial
012	54	high	1	serous	1st line treatment		no	no mutation	trial
013	32	high	1	mixed epithelial	relapse		no	no mutation	trial
014	50	high	3	serous	1st line treatment	BC 36	yes	BRCA1 c.3607C>T_p.Arg1203X	trial
015	56	high	4	serous	relapse		no	no mutation	
016	74	high	3	serous	1st line treatment	BC 54	no	no mutation	chemo choice
017	79	high	3	serous	remission	BC 77	no	no mutation	
018	51	high	3	serous	relapse		no	no mutation	trial
019	52	high	3	serous	relapse		no	no mutation	
020	52	high	3	serous	remission (later relapsed)		no	no mutation	trial
021	49	high	1	serous	remission		no	no mutation	
022	64	high	3	serous	1st line treatment		no	no mutation	
023	60	high	3	serous	relapse		no	no mutation	trial
024	52	high	3	serous	1st line treatment		no	no mutation	chemo choice
025	62	high	3	serous	relapse		no	no mutation	trial
026	46	high	3	serous	relapse		no	no mutation	chemo choice
027	50	high	3	serous	remission		no	no mutation	
028	49	high	2	endometroid	remission	EC synchronous	no	BRCA2 c.5279C>G_p.Ser1760X	breast surveillance
029	57	high	3	serous	remission (later relapsed)		no	no mutation	trial

ID	Age at OC	Grade	Stage	Histology	Timing of testing	Other cancer	Family History of BC/OC	Mutation status	How test influenced management
030	65	high	3	serous	1st line treatment		no	no mutation	trial
031	44	high	3	serous	relapse		no	no mutation	trial
032	53	high	3	endometroid	remission		no	no mutation	
033	36	high	4	serous	relapse		no	no mutation	trial
034	53	high	3	serous	relapse		yes	BRCA1 c.5266dupC	trial + breast surveillance
035	67	high	3	serous	relapse		no	no mutation	chemo choice
036	55	high	3	serous	relapse		yes	BRCA2 c.4398_4402delACATT	trial + breast surveillance
037	64	high	3	serous	remission (later relapsed)		no	no mutation	chemo choice
038	58	high	2	endometroid	remission		no	no mutation	
039	52	high	1	endometroid	remission		no	no mutation	
040	53	high	3	serous	1st line treatment		no	no mutation	
041	32	high	3	serous	1st line treatment		no	no mutation	trial
042	56	high	1	serous	relapse		no	no mutation	chemo choice
043	60	high	3	serous	relapse		no	no mutation	trial
044	64	high	4	serous	1st line treatment		no	no mutation	
045	58	high	4	serous	relapse		no	no mutation	
046	48	high	3	serous	remission (later relapsed)		no	no mutation	trial
047	55	high	3	serous	1st line treatment		no	no mutation	
048	51	high	1	endometroid	1st line treatment	EC synchronous	yes	no mutation	
049	46	high	3	serous	1st line treatment		no	BRCA2 c.6602delC	trial + breast surveillance
050	58	high	3	serous	1st line treatment		no	no mutation	trial
051	36	high	3	serous	1st line treatment		no	no mutation	
052	63	high	3	serous	relapse		no	no mutation	trial
053	46	high	3	serous	relapse		no	no mutation	chemo choice
054	47	high	3	serous	remission		no	no mutation	
055	47	high	4	serous	relapse		no	no mutation	trial
056	43	high	3	serous	remission (later relapsed)		no	BRCA2 deletion exon 14-16	breast surveillance
057	60	high	3	serous	1st line treatment		no	no mutation	
058	52	high	1	endometroid	relapse		no	no mutation	
059	64	high	4	serous	1st line treatment	BC 56	no	no mutation	trial
060	51	high	2	serous	relapse		no	no mutation	trial

ID	Age at OC	Grade	Stage	Histology	Timing of testing	Other cancer	Family History of BC/OC	Mutation status	How test influenced management
061	47	high	1	endometroid	remission		no	no mutation	
062	65	high	4	serous	1st line treatment		yes	BRCA2 c.5578A>T_p.Lys1860X	trial + breast surveillance
063	22	intermediate	3	serous	1st line treatment		no	no mutation	trial
064	55	high	3	serous	1st line treatment		no	BRCA2 c.7976+1G>A	breast surveillance
065	51	high	1	serous	remission		no	no mutation	
066	62	high	3	serous	relapse		yes	no mutation	chemo choice
067	47	high	3	serous	1st line treatment		no	BRCA1 c.181T>G_p.Cys61Gly	trial + breast surveillance
068	51	high	4	serous	relapse		yes	no mutation	
069	59	high	1	endometroid	remission		no	no mutation	
070	53	high	3	serous	relapse		no	no mutation	trial
071	64	high	3	serous	relapse		no	no mutation	trial
072	61	high	3	endometroid	relapse		no	no mutation	
073	43	high	2	endometroid	1st line treatment	EC synchronous	no	no mutation	
074	63	high	2	serous	remission		no	no mutation	
075	61	high	3	serous	remission		yes	BRCA1 c.329dupA	breast surveillance
076	53	high	3	serous	1st line treatment		no	no mutation	trial
077	58	high	3	serous	1st line treatment		no	no mutation	trial
078	50	high	2	serous	remission		no	BRCA2 c.5645C>A_p.Ser1882X	breast surveillance
079	63	high	1	serous	remission		no	no mutation	
080	63	high	1	serous	remission		yes	no mutation	
081	51	high	2	endometroid	1st line treatment		yes	no mutation	trial
082	44	high	3	serous	relapse		no	BRCA1 deletion exon 5-7	trial + breast surveillance
083	53	high	3	endometroid	relapse		no	no mutation	
084	64	high	3	serous	1st line treatment		no	no mutation	
085	49	high	3	serous	remission		no	no mutation	
086	64	high	3	endometroid	remission		no	no mutation	
087	49	high	4	serous	1st line treatment		yes	no mutation	trial
088	55	high	4	serous	relapse		no	BRCA1 c.3904G>T_p.Glu1302X	trial + breast surveillance
089	55	high	3	serous	relapse		no	BRCA1 c.3331_3334delAAG	trial + breast surveillance
090	59	high	3	serous	relapse		no	no mutation	trial
091	64	high	3	serous	1st line treatment		no	no mutation	trial
092	50	high	4	serous	relapse		yes	no mutation	

ID	Age at OC	Grade	Stage	Histology	Timing of testing	Other cancer	Family History of BC/OC	Mutation status	How test influenced management
093	58	high	4	serous	1st line treatment		no	no mutation	trial
094	55	high	3	serous	remission		no	no mutation	
095	53	high	1	serous	remission		no	no mutation	
096	67	high	4	serous	relapse	BC 61	no	no mutation	
097	47	high	3	serous	remission (later relapsed)		no	no mutation	trial
098	51	high	3	serous	relapse		no	no mutation	trial
099	54	high	3	serous	relapse		yes	BRCA1 c.5266dupC	trial + breast surveillance
100	59	high	3	serous	1st line treatment		no	no mutation	trial
101	64	high	3	serous	relapse		no	no mutation	
102	56	high	1	serous	remission		no	no mutation	
103	59	high	4	serous	relapse		no	no mutation	
104	62	high	3	serous	relapse		no	no mutation	trial
105	54	high	3	serous	remission		no	BRCA2 c.8167G>C_p.Asp2723His	breast surveillance
106	30	low	3	serous	relapse		no	no mutation	
107	51	high	1	serous	remission		no	no mutation	
108	62	high	3	serous	relapse		no	no mutation	trial
109	63	high	2	endometroid	remission	EC synchronous	no	no mutation	
110	64	high	3	serous	relapse		no	no mutation	trial
111	64	high	3	serous	remission	EC synchronous	no	BRCA2 c.5279C>G_p.Ser1760X	breast surveillance
112	59	high	1	endometroid	remission	EC synchronous	no	no mutation	
113	64	high	4	serous	remission	EC synchronous	no	no mutation	
114	69	high	3	serous	1st line treatment	BC 63	no	no mutation	chemo choice
115	47	high	2	endometroid	remission	EC synchronous	no	no mutation	
116	64	high	3	serous	1st line treatment	melanoma 49	no	BRCA2 c.4478_4481delAAAG	trial + breast surveillance
117	48	high	3	serous	remission		no	no mutation	
118	57	high	3	serous	remission		no	no mutation	
119	48	high	3	serous	relapse		no	no mutation	
120	54	high	3	serous	1st line treatment		yes	BRCA1 c.3607C>T_p.Arg1203X	trial + breast surveillance
121	58	high	3	serous	1st line treatment		no	no mutation	trial
122	64	high	3	serous	relapse		no	no mutation	trial
123	43	high	1	mixed epithelial	remission		no	no mutation	
124	38	high	3	serous	relapse		no	no mutation	trial

ID	Age at OC	Grade	Stage	Histology	Timing of testing	Other cancer	Family History of BC/OC	Mutation status	How test influenced management
125	68	high	4	serous	relapse		no	no mutation	trial
126	55	high	3	serous	relapse		no	no mutation	trial
127	57	high	3	serous	relapse		no	no mutation	
128	45	high	3	serous	remission		no	BRCA2 c.3975_3978dupTGCT	breast surveillance
129	64	high	3	serous	remission		no	no mutation	
130	57	high	1	serous	remission		no	no mutation	
131	45	high	3	serous	1st line treatment		no	BRCA1 c.2933dupA	trial + breast surveillance
132	52	high	3	endometroid	remission		no	no mutation	
133	56	high	3	serous	1st line treatment		no	no mutation	trial
134	48	low	4	serous	1st line treatment		yes	no mutation	trial
135	59	high	4	serous	1st line treatment		no	no mutation	trial
136	56	low	3	serous	remission		yes	no mutation	
137	52	high	3	serous	relapse		no	no mutation	trial
138	69	high	3	serous	relapse	EC synchronous	no	no mutation	trial
139	76	high	4	serous	1st line treatment		yes	BRCA1 deletion exon 1-17	breast surveillance
140	76	high	4	serous	relapse		no	no mutation	
141	55	high	1	endometroid	relapse		no	no mutation	chemo choice
142	63	high	3	serous	relapse		no	no mutation	trial
143	87	high	4	serous	relapse		no	BRCA2 c.9648+1G>T	trial + breast surveillance
144	54	high	3	serous	1st line treatment		no	no mutation	trial
145	56	high	3	serous	1st line treatment		yes	BRCA2 c.3458delA	breast surveillance
146	61	high	3	serous	relapse		no	no mutation	chemo choice
147	47	high	2	endometroid	1st line treatment		no	no mutation	
148	68	high	3	serous	relapse	BC 67, melanoma 31	no	no mutation	trial
149	60	high	2	serous	relapse		yes	BRCA1 c.1687C>T_p.Gln563X	trial + breast surveillance
150	47	high	3	serous	1st line treatment		yes	BRCA1 c.1360_1361delAG	trial + breast surveillance
151	53	high	2	serous	relapse	DCIS 50	yes	no mutation	
152	80	high	4	serous	relapse		no	no mutation	
153	64	high	4	serous	remission		no	no mutation	
154	54	high	1	serous	relapse		no	no mutation	trial
155	60	high	3	serous	1st line treatment		yes	no mutation	trial

ID	Age at OC	Grade	Stage	Histology	Timing of testing	Other cancer	Family History of BC/OC	Mutation status	How test influenced management
156	53	high	3	serous	remission	BC 40	no	BRCA1 c.5266dupC	breast surveillance
157	53	low	4	serous	1st line treatment		no	no mutation	trial
158	47	high	4	serous	relapse		no	no mutation	trial
159	63	high	3	serous	1st line treatment		no	no mutation	trial
160	79	high	4	serous	1st line treatment	BC 54, 79	yes	no mutation	chemo choice
161	53	high	4	serous	relapse	CML 43	no	no mutation	trial
162	66	high	3	serous	remission		no	no mutation	
163	52	high	3	serous	remission		yes	BRCA1 c.4527C>G_p.Tyr1509X	breast surveillance
164	59	high	4	serous	1st line treatment		yes	no mutation	trial
165	71	high	3	serous	1st line treatment		no	no mutation	trial
166	63	high	3	serous	1st line treatment		no	no mutation	trial
167	76	high	3	serous	1st line treatment		no	no mutation	trial
168	68	high	4	serous	remission (later relapsed)		no	no mutation	trial
169	46	high	3	serous	relapse		no	no mutation	trial
170	77	high	4	serous	relapse	BC 48, 68	yes	no mutation	
171	68	high	3	serous	remission		no	no mutation	
172	67	high	3	serous	remission		no	no mutation	
173	81	high	4	serous	1st line treatment		no	no mutation	
174	63	high	3	serous	1st line treatment	BC 56	no	no mutation	
175	78	high	3	serous	relapse		no	no mutation	trial
176	46	high	2	endometroid	relapse		yes	no mutation	trial
177	67	high	3	serous	relapse		no	no mutation	trial
178	60	high	3	serous	1st line treatment		no	no mutation	trial
179	68	high	2	serous	1st line treatment		no	no mutation	
180	70	high	3	serous	1st line treatment		no	no mutation	trial
181	40	high	3	mixed epithelial	remission		yes	no mutation	
182	30	high	3	serous	1st line treatment		no	BRCA1 c.5266dupC	trial + breast surveillance
183	62	high	3	serous	1st line treatment		no	no mutation	trial
184	71	high	3	serous	relapse		no	no mutation	trial
185	79	high	1	endometroid	remission	BC 75	no	no mutation	
186	53	high	4	mixed epithelial	remission		no	no mutation	

ID	Age at OC	Grade	Stage	Histology	Timing of testing	Other cancer	Family History of BC/OC	Mutation status	How test influenced management
187	53	high	3	serous	remission		no	no mutation	
188	50	high	4	serous	remission		no	no mutation	
189	78	high	3	serous	1st line treatment		no	no mutation	trial
190	71	high	3	serous	relapse		no	no mutation	trial
191	48	high	3	serous	relapse		no	no mutation	chemo choice
192	53	high	2	clear cell	remission		yes	no mutation	
193	51	low	3	serous	relapse		no	no mutation	trial
194	66	high	3	serous	1st line treatment		no	no mutation	trial
195	63	high	1	serous	relapse		yes	no mutation	trial
196	68	high	3	serous	relapse		no	no mutation	trial
197	69	high	3	serous	1st line treatment		yes	no mutation	
198	75	high	3	serous	1st line treatment	BC 55	yes	no mutation	trial
199	58	high	3	serous	relapse		yes	no mutation	trial
200	68	high	1	serous	remission		no	no mutation	
201	50	high	3	serous	1st line treatment		no	no mutation	trial
202	50	high	3	serous	1st line treatment		yes	BRCA1 c.2612_2613insT	trial + breast surveillance
203	54	high	3	clear cell	1st line treatment		no	no mutation	chemo choice
204	75	high	4	serous	1st line treatment		no	no mutation	trial
205	62	high	3	serous	relapse		yes	BRCA2 c.2330dupA	
206	76	high	3	serous	1st line treatment		no	no mutation	trial
207	56	high	3	serous	1st line treatment		no	no mutation	trial

Key: **BC**: breast cancer, **OC**: ovarian cancer, **EC**: endometrial choice cancer, **DCIS**: ductal carcinoma in situ (breast), **CML**: chronic myeloid leukemia, **trial**: eligible for therapeutic trial, **chemo choice**: status impacted choice of chemotherapy, **breast surveillance**: eligible for enhanced breast surveillance

Supplementary Table 2. Summary of patient feedback

Key question response summaries

Question		Questionnaire v.1 no mut	Questionnaire v.2 no mut	Questionnaire v.1 Mutation	Questionnaire v.2 mutation	Totals	Total %
I was given a Patient Information Sheet prior to my test	Agree	44	10	42	4	**100**	**95%**
	Unsure	0	1	0	0	**1**	**1%**
	Disagree	0	0	3	1	**4**	**4%**
The written information provided was clear and helpful	Agree	43	10	43	5	**101**	**97%**
	Unsure	1	1	0	0	**2**	**2%**
	Disagree	0	0	1	0	**1**	**1%**
I was clear in my own mind why I was being offered a genetic test.	Agree	43	10	43	5	**101**	**98%**
	Unsure	1	1	0	0	**2**	**2%**
	Disagree	0	0	0	0	**0**	**0%**
I was given sufficient time to think about whether I wanted a test	Agree	42	10	43	5	**100**	**98%**
	Unsure	0	1	0	0	**1**	**1%**
	Disagree	1	0	0	0	**1**	**1%**
I was aware that I could have additional discussions with the Genetics team before deciding whether to have a test.	Agree	39	6	29	4	**78**	**76%**
	Unsure	3	4	7	0	**14**	**14%**
	Disagree	2	1	7	1	**11**	**11%**
I was aware that the result of the test might impact me.	Agree	43	11	42	5	**101**	**96%**
	Unsure	1	0	2	0	**3**	**3%**
	Disagree	0	0	1	0	**1**	**1%**
I was aware that the result of the test might have implications for my family.	Agree	44	11	43	5	**103**	**98%**
	Unsure	0	0	2	0	**2**	**2%**
	Disagree	0	0	0	0	**0**	**0%**
I was aware that further discussions with the Genetics Team would be organised for me if a gene mutation was found.	Agree	40	10	37	5	**92**	**88%**
	Unsure	3	1	6	0	**10**	**10%**
	Disagree	1	0	2	0	**3**	**3%**
I was informed when the result would be available.	Agree	39	9	39	5	**92**	**88%**
	Unsure	4	2	5	0	**11**	**10%**
	Disagree	1	0	1	0	**2**	**2%**
I was informed how I would receive the result.	Agree	35	7	41	4	**87**	**83%**
	Unsure	7	3	3	1	**14**	**13%**
	Disagree	2	1	1	0	**4**	**4%**
When I received my result I was given a Patient Information Sheet about receiving a normal BRCA1 and BRCA2 result	Agree	35		41		**76**	**86%**
	Unsure	3		1		**4**	**5%**
	Disagree	6		2		**8**	**9%**
I understood the implications of this result for me.	Agree	40		42		**82**	**92%**
	Unsure	3		3		**6**	**7%**
	Disagree	1		0		**1**	**1%**
When I received my result, I was told I would be sent an appointment to see the Genetics Team.	Agree		11			**11**	**100%**
	Unsure		0			**0**	**0%**
	Disagree		0			**0**	**0%**
When I received my result, I also received an appointment with the Genetics Team.	Agree				5	**5**	**100%**
	Unsure				0	**0**	**0%**
	Disagree				0	**0**	**0%**
My appointment with the Genetics team helped me to understand what the result meant for me and for my family.	Agree		11		5	**16**	**100%**
	Unsure		0		0	**0**	**0%**
	Disagree		0		0	**0**	**0%**
When I received my result, I also received a Patient Information Sheet about receiving a BRCA1 and BRCA2 test result that identifies a mutation.	Agree				5	**5**	**100%**
	Unsure				0	**0**	**0%**
	Disagree				0	**0**	**0%**
Within a month of receiving my result I had been sent a date for an appointment with the Genetics Team.	Agree		9			**9**	**82%**
	Unsure		2			**2**	**18%**
	Disagree		0			**0**	**0%**
I was happy to receive a copy of my test report (results).	Agree			44	5	**49**	**98%**
	Unsure			0	0	**0**	**0%**
	Disagree			1	0	**1**	**2%**
I was happy to receive my results by letter.	Agree			42	5	**47**	**96%**
	Unsure			0	0	**0**	**0%**
	Disagree			2	0	**2**	**4%**
I was happy to have the genetic test at one of my existing Oncology appointments rather than in a separate appointment with the Genetics Team.	Agree	43	11	45	4	**103**	**98%**
	Unsure	1	0	0	0	**1**	**1%**
	Disagree	0	0	0	1	**1**	**1%**
I am pleased I had the genetic test.	Agree	44	11	45	5	**105**	**100%**
	Unsure	0	0	0	0	**0**	**0%**
	Disagree	0	0	0	0	**0**	**0%**

Note: Not every respondee answered every Q - hence question summary totals <105):

See Supplementary Figure 2 for patient feedback questionnaire

Supplementary Table 3. Summary of cancer team feedback

Question	Average Score
I believe it is important for ovarian cancer patients to be able to benefit from BRCA gene testing.	5.0
There is increasing interest from ovarian cancer patients to have BRCA gene testing.	4.9
I welcome the opportunity to carry out BRCA gene testing for ovarian cancer patients through oncology appointments.	4.8
I found it helpful to have supporting materials (e.g. training materials and FAQs) containing information on carrying out BRCA gene testing.	4.8
It was useful to be able to complete the BRCA gene testing training materials at a time that was convenient for me.	4.4
It is useful to have an approved clinical protocol to follow when carrying out BRCA gene testing.	4.7
It is useful to have information sheets to provide to patients about BRCA gene testing.	4.6
I feel confident to consent a patient for a BRCA gene test.	4.3
It is possible to discuss BRCA gene testing with a patient within the timeframe of a consultation.	4.2
I was clear when the BRCA gene test result would be available.	4.0
The process for carrying out BRCA gene testing worked well.	4.2

Responses were scored as follows:
1=Strongly disagree 2=Disagree 3=Unsure 4=Agree 5=Strongly agree

Supplementary Appendix

Mainstream BRCA Testing Implementation Kit - Ovarian Cancer

Introduction
These resources have been developed to support implementation of BRCA testing through routine oncology appointments. We call this Mainstreaming Cancer Genetics (MCG).

These resources have been developed for use at the Royal Marsden. We hope that they will serve as a useful template for other hospitals wishing to implement a similar service.

Using these resources
Please read all the information in this implementation kit. Feel free to make amendments to tailor them for your own service.

Please also view the e-learning modules on the MCG YouTube channel: www.youtube.com/mcgprogramme. You may wish to use these videos as part of your own training programme, particularly modules MCG ELM1 and ELM2, or to use them as a template for developing your own materials.

Please keep up to date with MCG programme outputs through our website. Sign up at www.mcgprogramme.com (click 'Follow' in the bottom right corner of the screen).

Contents
The implementation kit includes the following:

- MCG P2 - Ovarian cancer BRCA testing protocol
- MCG IS1 - BRCA1 and BRCA2 gene testing - Information sheet for patients with cancer
- MCG IS2 - Receiving a normal BRCA1 and BRCA2 test result - Information sheet for patients with cancer
- MCG IS3 - Receiving a BRCA1 and BRCA2 test result that identifies a mutation - Information sheet for patients with cancer
- MCG IS4 - Receiving a BRCA1 and BRCA2 test result that identifies a variant requiring evaluation (VRE) - Information sheet for patients with cancer
- MCG F1 - Consent for genetic testing
- MCG FAQ1 - BRCA1 and BRCA2 gene testing - frequently asked questions for breast and gynae units

Additional resources for genetics teams:

- MCG GU SOP1 - Cancer Genetic Unit standard operating procedure for returning results
- MCG GRL1 - Template genetics results letter – No mutation identified
- MCG GRL2 - Template genetics results letter – Mutation identified
- MCG GRL3 - Template genetics results letter – Variant requiring evaluation identified

Acknowledging the MCG programme
We have made these resources freely available to assist other hospitals in developing mainstream gene testing services. We would be grateful if you could let us know if you use them, and that you acknowledge us.

If these resources are modified and used in any publications, presentations or other communications, we request that the following acknowledgement is included:

'We acknowledge the support of the Mainstreaming Cancer Genetics (MCG) programme (www.mcgprogramme.com), in developing these resources'.

Ovarian cancer BRCA testing protocol

Cancer Team

Patient with non-mucinous ovarian cancer

↓

Actions by approved Cancer Team member:
1. Information sheet (MCG IS1) given to patient.
2. BRCA gene testing discussed.
3. Consent (MCG F1) obtained and scanned onto EPR.
4. Blood (2xEDTA) and request form (MCG F2) sent to lab.

More discussion required → Refer to Genetics

↓

Genetics Team

Result reviewed and interpreted by Genetics Team

↓

**NO MUTATION**	**MUTATION**	**VARIANT REQUIRING EVALUATION (VRE)**
Actions by Genetics:<br>1. Result and information sheet (MCG IS2) sent to patient.<br>2. Result sent to Cancer Team.	Actions by Genetics:<br>1. Result and information sheet (MCG IS3) sent to patient.<br>2. Result sent to Cancer Team.<br>3. Genetics appt sent to patient.	Actions by Genetics:<br>1. Result and information sheet (MCG IS4) sent to patient.<br>2. Result sent to Cancer Team.<br>3. Genetics appt sent to patient.

Notes:
For FAQs (MCG FAQ1) see: www.mcgprogramme.com/BRCAtesting

BRCA1 and BRCA2 gene testing

Information sheet for patients with cancer

In most people cancer occurs by chance. In a minority of people with ovarian cancer (about 15%) or breast cancer (about 3%), cancer occurs because they have a mutation in the BRCA1 or BRCA2 gene.

BRCA1 and BRCA2 mutations result in increased risks of breast and ovarian cancer. They occur more frequently in women who have both breast and ovarian cancer, those with particular types of cancer, and if there is a strong family history of breast and/or ovarian cancer. It is important to identify if a cancer is due to a BRCA1 or BRCA2 mutation because it provides you and your doctors with information that can help treat your cancer and to reduce your risk of future cancer. It can also provide information for relatives about their risks of cancer.

Why am I being offered this test?
You are being offered a test to look for mutations in BRCA1 and BRCA2 because of your cancer diagnosis.

What are the benefits to me?
Knowing whether or not you carry a mutation in BRCA1 or BRCA2 gives the cancer team more information about your cancer. This can help decisions about the treatments they recommend for you, for example which chemotherapy drugs or surgery would be most suitable. It will also give better information about your risk of developing cancer in the future.

Does having the test have implications for my family?
In most people the test will be normal and we will not find a gene mutation. This would be reassuring for relatives as it would indicate that your cancer was unlikely to be due to hereditary factors that would put them at very high risk of cancer. If your test shows you have a gene mutation, it is possible that some relatives also have the mutation. Relatives would be able to discuss this with a specialist geneticist and have a test if they chose to.

What will happen if NO mutation in BRCA1 or BRCA2 is found?
This is the most likely outcome, as most women with cancer do not have a mutation in BRCA1 or BRCA2. This would be reassuring in suggesting you are unlikely to be at high risk of developing another, new cancer in the future. The cancer team will be able to use this information in their management decisions. Very occasionally mutations in other genes can be involved in causing breast or ovarian cancer. Also new discoveries are being made all the time. If a new gene test becomes available in the future the genetics team may be able to do the test using the sample you have already provided and would send the result to you and the cancer team. If your doctors think other genetic factors might be involved in your cancer they can ask the genetics clinic to send you an appointment to evaluate this, if you have not already had an appointment with genetics.

What will happen if a BRCA1 or BRCA2 mutation is found?
Your cancer team will use the information in their management decisions. The genetics team will send you an appointment to discuss the results and address any questions you have. They will also discuss what the test result means for your future risk of cancer, your options for future screening and measures to reduce these risks. They will evaluate your family history and can provide information for the appropriate family members should they wish to consider testing to see if they have inherited the mutation. Any relatives can be referred to a Genetics Unit, either at the Royal Marsden or more locally to them, to discuss this further.

What will happen if the test result is unclear?
Very occasionally (<1%) we find a gene change, known as a 'variant', that needs further assessment before we can decide if it is linked to why you have had cancer. If this occurs, the genetics team will send you an appointment to explain the result and to discuss with you what further information and/or tests would be helpful to find out if the variant is linked to your cancer.

Do I have to have the test?
No, having this test is optional. Your decision will not affect the standard of care you receive from the hospital or doctor, which will be based on the available information.

What if I am not sure if I want to have the test?
We would recommend for you to have further discussions with a specialist member of the genetics team.

What will happen next if I say yes?
If you decide to have the test, you will be asked to sign a consent form. A blood sample will be taken for the test.

How will I receive the results of the test?
The genetics team will send you and your cancer team the results of the test by post. The results may take up to 8 weeks, but will usually be within 4-6 weeks.

Will my information be confidential?
All data collected about you will be held under the provisions of the 1998 Data Protection Act and stored in secure files. The only people who will know your identity are the hospital staff and a few trained staff reporting the results who are bound by a professional duty to protect your privacy.

If you have any questions please contact the Royal Marsden Cancer Genetics Unit on 0207 661 3375 or cancergenetics@rmh.nhs.uk

Receiving a normal BRCA1 and BRCA2 test result

Information sheet for patients with cancer

You had a BRCA1 and BRCA2 gene test because you have had cancer.
The test result is normal.
No BRCA1 or BRCA2 mutation (gene change) was identified in your blood sample.

What does this result mean for me?
This means we have not found a BRCA1 or BRCA2 mutation which would put you at high risk of developing another cancer. The cancer team will discuss if this normal result has any implications for your cancer management.

A normal result is common. In most women with breast and/or ovarian cancer no mutation in BRCA1 or BRCA2 is found. If you have a strong family history of breast and/or ovarian cancer, or a strong family history of other cancers, or if you developed cancer at an unusually young age, it may be helpful to look into things further. The genetics or cancer team will discuss this with you, if appropriate.

Very occasionally mutations in other genes can be involved in causing breast or ovarian cancer. Also new discoveries are being made all the time. If a new gene test becomes available the genetics team may be able to do the test using the sample you have already provided and they will send the result to you and your cancer team.

What does this result mean for my relatives?
This result is good news for your relatives, as it means they are unlikely to be at high increased risk of developing breast and/or ovarian cancer themselves. You may wish to share this result with them.

All women are eligible to have mammograms from 47 years in the National Breast Screening Programme. Depending on the family history, some women may be eligible for mammograms from 40 years, even if there has been a normal BRCA1 or BRCA2 gene test in the family. There is currently no known effective form of ovarian screening. If a woman has multiple relatives with ovarian cancer removal of the ovaries is sometimes considered.

If any of your relatives wish to discuss their own risks of cancer further they should speak with their GP who can refer them for further discussions at a Family History or Genetics clinic.

If you have any further questions, please contact the Royal Marsden Cancer Genetics Unit on 0208 661 3375 or cancergenetics@rmh.nhs.uk

Receiving a BRCA1 and BRCA2 test result that identifies a mutation

Information sheet for patients with cancer

You had a BRCA1 and BRCA2 gene test because you have had cancer.

The test result has shown that you have a mutation (gene change) in either the BRCA1 or BRCA2 gene. The exact details of the mutation are given in the test report.

BRCA1 or BRCA2 mutations result in increased risks of breast and ovarian cancer, and occasionally other cancers. Therefore this result provides an explanation for why you developed cancer.

Your cancer team will discuss with you if this result has implications for your cancer treatment and/or follow-up.

This result has implications for your future health and potentially for your relatives. An appointment has been made for you in the Genetics clinic to discuss these issues further. At the appointment you will be able discuss your future risks of cancer and your options for cancer screening and measures to reduce the risk of cancer. The potential implications for relatives will also be discussed. The processes by which your relatives can have discussions themselves to decide if they wish to have testing will be explained.

You may find it helpful to read the information booklet “A Beginner’s Guide to BRCA1 and BRCA2” which gives more detailed information. This can be downloaded from www.royalmarsden.nhs.uk/brca

If you need to discuss anything urgently prior to your appointment, or wish to alter the date of your appointment, please contact the Royal Marsden Cancer Genetics Unit on 0208 661 3375 or cancergenetics@rmh.nhs.uk

Receiving a BRCA1 and BRCA2 test result that identifies a variant requiring evaluation (VRE)

Information sheet for patients with cancer

You had a BRCA1 and BRCA2 gene test because you have had cancer.

The test result has shown that you have a gene change (variant) in either the BRCA1 or BRCA2 gene that requires further evaluation.

At the moment, we do not have enough information to decide if this variant is linked to why you have had cancer.

Variants in the BRCA1 and BRCA2 genes are common, and most do not cause cancer. Very occasionally, we find a variant that requires further assessment before we can decide if it leads to an increased risk of cancer. In some cases, we may need to do further blood tests to help us find out more about the impact of the variant.

We have made an appointment for you in the Genetics clinic to discuss your result further. At the appointment we will explain in more detail about the result and any further tests that may be required. We will also discuss the process and timeframe for deciding if the variant is likely to be linked to your cancer.

It is important for us to have as much information as possible when we see you. We have enclosed a family history questionnaire with your letter, and would be very grateful if you would fill this in and return it to us before we see you in clinic.

If you need to discuss anything urgently prior to your appointment, or wish to alter the date of your appointment, please contact the Royal Marsden Cancer Genetics Unit on 0208 661 3375 or cancergenetics@rmh.nhs.uk

Consent for Genetic Testing

**THE ROYAL MARSDEN NHS FOUNDATION TRUST**<br><br>**Patient / parental agreement to investigation**	Patient's surname/family name
	Patient's first names
	Date of birth
	Responsible health professional
	Job title
	NHS number (or other identifier)
**See overleaf for:**<br>**(1) Special Requirements (2) Information provided and (3) Consent policy**	☐ Male ☐ Female

Statement of health professional (to be filled in by health professional with appropriate knowledge of proposed procedure, as specified in consent policy)

The purpose of these investigations is to help establish the causes of cancer and/or risk of cancer for you. The results may also provide information which may be helpful for other family members. We have provided written information outlining the risks and benefits of these investigations. During the consultation and/or through written information we have discussed the following issues related to gene testing and you have agreed to the numbered statements indicated below. If you have further questions please contact the genetics team on **0208 661 3375** or **The Royal Marsden, Cancer Genetics Unit, Downs Road, Sutton, SM2 5PT**

Please circle as appropriate

1	I agree to the testing of ……………………………………………………	Yes		
2	I understand that the test results will be put on the electronic patient records at the Royal Marsden NHS Foundation Trust.	Yes		
3	I understand the sample will be stored in case new gene tests become available.	Yes		
4	I understand that additional tests may be undertaken on the stored sample, if indicated, and I will be informed of any relevant results.	Yes		
5	I agree that the test results can be made available to doctors looking after family members, on request.	Yes	No	N/A
6	I agree to the sample being used anonymously for research.	Yes	No	
**Additional issues discussed**:				

Signature: ___________________________ Print name: __________________________ Date: _____________
(Patient / Parent / Guardian / Relative)

To be completed if individual identified above is deceased:
I agree to the above genetic tests and sharing of information on behalf of my relative.
Relationship to patient: ________________________

Signature: ___________________________ Print name: __________________________ Date: _____________
(Clinician)

The following information leaflet / consultation letter(s) have been provided:

ÿ ……………………………………….……….…… ………………….. (version no._________)

ÿ ……………………………………………….…………………………… (version no._________)

Special requirements (e.g. other language/other communication method):

……………………………………………………………………………………………………..

……………………………………………………………………………………………………..

Guidance to health professionals (to be read in conjunction with consent policy)

This form

This form documents the patient's agreement (or that of a person with parental responsibility for the patient) to go ahead with the investigation you have proposed**. It is only designed for use where the patient is expected to remain alert throughout and where an anaesthetist is not involved in their care.**

Consent forms are not legal waivers – if patients, for example, do not receive enough information on which to base their decision, then the consent may not be valid, even though the form has been signed. Patients also have every right to change their mind after signing the form.

Who can give consent

Everyone aged 16 or more is presumed to be competent to give consent for themselves, unless the opposite is demonstrated. If a child under the age of 16 has "sufficient understanding and intelligence to enable him or her to understand fully what is proposed", then he or she will be competent to give consent for himself or herself. Young people aged 16 and 17, and legally 'competent' younger children, may therefore sign this form for themselves, if they wish. If the child is not able to give consent for himself or herself, some-one with parental responsibility may do so on their behalf. Even where a child is able to give consent for himself or herself, you should always involve those with parental responsibility in the child's care, unless the child specifically asks you not to do so. If a patient is mentally competent to give consent but is physically unable to sign a form, you should complete this form as usual, and ask an independent witness to confirm that the patient has given consent orally or non-verbally.

When NOT to use this form (see also 'This form' above)

If the patient is 18 or over and lacks the capacity to give consent, you should use form 4 (form for adults who lack the capacity to consent to investigation or treatment) instead of this form. A patient lacks capacity if they have an impairment of the mind or brain or disturbance affecting the way their mind or brain works and they cannot:

- understand information about the decision to be made
- retain that information in their mind
- use or weigh that information as part of the decision-making process, or
- communicate their decision (by talking, using sign language or any other means).

You should always take all reasonable steps (for example involving more specialist colleagues) to support a patient in making their own decision, before concluding that they are unable to do so.
Relatives **cannot** be asked to sign a form on behalf of an adult who lacks capacity to consent for themselves, unless they have been given the authority to so under a Lasting Power of Attorney or as a court appointed deputy.

Information

Information about what the investigation will involve, its benefits and risks and the alternatives to the particular test proposed, is crucial for patients when making up their minds about investigations. The courts have stated that patients should be told about 'significant risks which would affect the judgement of a reasonable patient'. 'Significant' has not been legally defined, but the GMC requires doctors to tell patients about 'serious or frequently occurring' risks. In addition if patients make clear they have particular concerns about certain kinds of risk, you should make sure they are informed about these risks, even if they are very small or rare. You should always answer questions honestly. Sometimes, patients may make it clear that they do not want to have any information about the options, but want you to decide on their behalf. In such circumstances, you should do your best to ensure that the patient receives at least very basic information about what is proposed. Where information is refused, you should document this overleaf or in the patient's notes.

The law on consent

See the Department of Health's *Reference guide to consent for examination or treatment* for a comprehensive summary of the law on consent (also available at www.dh.gov.uk/consent).

BRCA1 and BRCA2 gene testing

Frequently asked questions for breast and gynae units

MCG FAQ1 - last updated 09/02/15

Q: Which patients can have BRCA testing through oncology?
Non-geneticists can only undertake BRCA testing (i.e. to look in blood for mutations in the BRCA1 and BRCA2 genes) in breast cancer (BC) or ovarian cancer (OC) patients that fulfill one or more of the following criteria (see Protocol MCG P1 and Protocol MCG P2).

NHS RM patient with

- Non-mucinous OC diagnosed at any age
- BC <40 years
- Bilateral BC, both <60 years
- Triple-negative BC, any age
- BC + OC, any age
- Male BC, any age
- Any BC patient in whom gene testing would impact current cancer management

Other patients, such as those with a family history of cancer who do not fulfill any of the above criteria may be eligible for testing, either through NHS or through research, but will need to be referred to Genetics by email and/or letter (not by EPR) for this to be evaluated and performed if appropriate. Referrals and queries about whether or not patients are eligible should be directed to cancergenetics@rmh.nhs.uk.

Q: Which non-geneticists can perform BRCA testing in eligible patients?
Only non-geneticists that have completed the 30 min online training can perform BRCA testing. Details about the training are available at www.mcgprogramme.com. Contact cancergenetics@rmh.nhs.uk if you wish to become approved to perform BRCA testing.

Q: How were the eligibility criteria for ovarian cancer patients decided?
For ovarian cancer the eligibility criteria are in line with the NICE recommendations www.nice.org.uk/guidance/cg164 which state that any patient with ≥10% chance of having a BRCA mutation should be tested. Several studies, and our own RM data, have shown that any non-mucinous ovarian cancer patient meets this threshold.

Q: How were the eligibility criteria for breast cancer patients decided?
When we introduced the mainstreaming programme for breast cancer patients we used eligibility criteria we believed would be at ~10% mutation detection threshold, however the mutation detection rate after the first year was 20%. Moreover the economic modelling for the NICE guidelines indicated that testing was cost-effective at 5% threshold but this was not formally recommended because of limited Clinical Genetic capacity. We therefore decided to change the criteria to increase testing access. The current testing eligibility criteria are being evaluated as part of a two year study funded by the NIHR RM/ICR Specialist BRC.

Q: Who is leading the Mainstreaming Cancer Genetics BRCA initiative?
The initiative is part of the Mainstreaming Cancer Genetics (MCG) programme. Professor Nazneen Rahman is Programme Director and Drs Helen Hanson, Zoe Kemp and Angela George are on the Programme board. All are also Consultant Cancer Geneticists at the Royal Marsden. You can read more about the initiative at www.mcgprogramme.com.

Q: Why is there a testing category for BC patients in whom testing would impact cancer management?
Traditionally eligibility for BRCA testing was decided by estimating the chance that a mutation would be detected. However, over the last 5 years it has become apparent that BRCA testing can impact on treatment options and increasingly patients and cancer doctors have been requesting testing to help with management decisions. Through the study we wish to evaluate the reasons why BRCA testing is requested, the mutation detection rates in groups tested for treatment decisions, and the impact of the testing for the patient.

Q: Which BC patients should I offer BRCA testing to on the basis that it would impact current cancer management?
For the first six months of the pilot we are operating an open-door policy such that you can use your own judgement and offer testing to anyone that you feel it would be helpful. We only ask that you document the reason so that we can perform evaluations and determine the mutation detection rate in different scenarios. After six months we will review the data and may stipulate specific cancer management criteria for testing.

Q: Where do I record the reason for carrying out BRCA testing for BC patients tested because it would impact current cancer management?
Please document the reason on the lab referral form (MCG F2) in the space provided.

Q: What is the testing eligibility for private patients?
Any private patient with breast or ovarian cancer can have BRCA testing if they have been consented by an approved clinician. A specific process for this has been set-up and is available in Private Care, or from rahmanRMPC@icr.ac.uk.

Q: What about one-off second opinions?
Patients that are seen as one-off second opinions are currently excluded from BRCA testing through the RM breast and gynae units because the processes for return of results, follow-up and reimbursement are not in place. We are looking into how we might address the needs of these patients.

Q: How strict are the age cut-offs for testing?
These are strictly applied. For example, a woman with bilateral breast cancer diagnosed at 58 years and 60 years (and no family history) would not be eligible for testing, but if she were diagnosed at 58 years and 59 years she would be eligible. It is recognised, and inevitable, that individuals close to a threshold may have similar likelihoods of carrying a mutation but different eligibility. We are working hard to make eligibility generally more permissive, but in the meantime it is important for clinicians and patients to have confidence that criteria are being consistently applied.

Q: How is triple-negative breast cancer defined?
For the purpose of eligibility for genetic testing a triple-negative breast cancer is defined as a tumour with an Allred score of 0, 1 or 2 for ER and PR receptors and HER2 negativity by IHC or FISH.

Q: Should *in situ* breast cancer be included?
In situ cancer, such as DCIS (ductal carcinoma in situ) and LCIS (lobular carcinoma in situ), should be included in the same way as invasive breast cancer in assessing eligibility for BRCA testing.

Q: How should multiple metachronous ipsilateral breast cancers be assessed?
Two (or more) separate, ipsilateral breast cancers which have occurred 5 or more years apart should be considered as separate cancers (i.e. counted as a bilateral breast cancer) when assessing eligibility for BRCA testing, unless it is clear that the second cancer is a

recurrence. This is a pragmatic approach as it is not possible to robustly identify which are separate cancers and which recurrence.

Q: How should multiple synchronous ipsilateral breast cancers be assessed?
Simultaneous ipsilateral breast cancers, whether termed synchronous, multicentric or multifocal, should be counted as a single breast cancer for assessing eligibility for BRCA testing.

Q: What is the BRCA testing eligibility for Ashkenazi Jewish patients?
Exactly the same eligibility criteria apply for Ashkenazi Jewish patients, who will have full gene testing, not just testing for the three Ashkenazi founder mutations.

Q: When should discussion of BRCA testing be undertaken?
This should be at the discretion of the clinician. BRCA testing can be discussed and undertaken at the time of diagnosis, during active cancer management or during follow up. However, please be aware that the result takes ~4 weeks from receipt of sample, so if the result is required for management decisions, timing of testing must be planned accordingly.

Q: What information should I give to the patient prior to obtaining consent?
The information sheet 'BRCA1 and BRCA2 gene testing - Information sheet for patients with cancer' (MCG IS1) should be given to the patient. Patients should be informed that BRCA mutations are a cause of cancer and knowing whether or not a BRCA mutation is involved in causing their cancer can be helpful for their current and future management. The clinician may like to describe the specific relevance of the test for the specific patient. The patient should also be aware that the result can provide information of relevance to the wider family. However, it is important to remember that most tests are normal and therefore detailed discussions regarding risk management for patient and relatives, prior to testing, are not required. If a patient has questions that require either more time or more expertise than you are able to provide, the patient should be referred to Genetics. E-learning modules 1 (MCG ELM1) and 2 (MCG ELM2) provide further information about BRCA testing.

Q: What are the insurance implications for cancer patients?
If a cancer patient applies for life cover, critical illness or income protection cover after the gene test is performed then it will need to be disclosed, along with the other information about their cancer diagnosis. This is unlikely to have impact on the cover/terms they are offered over and above the impact of their cancer diagnosis. If the gene test was performed after an insurance policy was set-up the result does NOT need to be disclosed.

Q: Are there insurance implications for the cancer patients relatives?
Relatives would need to tell the insurance company about the cancer diagnosis and if a gene mutation has been found when asked about their family history (if they are aware of it). If the test is normal some insurance companies may take this into consideration to mitigate the unfavourable impact of the family history on the policy. Unaffected individuals do not have to disclose the results of predictive gene testing to insurance companies but may choose to do so, particularly if the test is negative.

Q: Which consent form should I use?
You should use the 'Consent for Genetic Testing' form (MCG F1) which is available on EPR. The form is also available online at www.mcgprogramme.com/BRCAtesting. E-learning module 4 (MCG ELM4) explains how to take consent. Once completed, the form should be scanned onto EPR and a copy given to the patient. It should NOT be sent to the lab.

Q: Which lab form should I use?
There is a specific lab form 'BRCA gene test request form' (MCG F2). The form will be available in clinics and online at www.mcgprogramme.com/BRCAtesting. Please send the form to the TGL clinical lab in BLB in ICR, together with the sample (2x9ml EDTA).

Q: How long does it take to get a BRCA result?
The results of full analysis of the BRCA1 and BRCA2 genes typically take about 4 weeks, but may take up to 6 weeks. If there is a known mutation in the family the result takes 2-4 weeks.

Q: How is the BRCA testing being done?
We are using a next-generation sequencing panel called the TruSight Cancer panel, which has undergone extensive validation. All mutations are further confirmed by Sanger sequencing and/or MLPA as appropriate. Further details of the panel are available at www.mcgprogramme.com.

Q: Will we receive results of only the BRCA genes?
Yes. At the current time we are reporting just the results of the BRCA1 and BRCA2 genes. We will be extending to additional genes that cause breast and/or ovarian cancer in the near future.

Q: What if a patient meets the criteria but chooses not to have a test?
The test is optional. A patient may decline to be tested, ask to have longer to think about testing or be referred to Genetics if they want, or need, more detailed discussions.

Q: What if a patient does not meet the testing criteria but wants a test?
Patients who do not meet the mainstreaming testing criteria may be eligible for a test on account of their family history. If this seems possible they should be referred to Genetics. Please note, acceptance of the referral by Genetics does not indicate that testing will definitely be performed. Queries about whether or not patients are eligible should be directed to cancergenetics@rmh.nhs.uk. Alternatively they could choose to have the test privately.

Q: What if a family member has already had BRCA testing?
If a member of the family has already had a BRCA test, please contact the Genetics unit on cancergenetics@rmh.nhs.uk and note this on the lab form. It may influence the testing that is performed.

Q: Who will give the patient the result of the BRCA test?
The Genetics team will write to the patient with the result and will send an information sheet with additional information. The referring clinician and GP will also be notified. The result will be uploaded to the Germline Genetics tab on EPR together with an EPR annotation of the result.

Q: What happens if no mutation is identified?
The Genetics team will inform the patient of the result in writing and will send the patient a copy of the report and the information sheet 'Receiving a normal BRCA1 and BRCA2 test result' (MCG IS2). You should use the result as appropriate for their cancer management, but usually no further input is required from Genetics. If the patient has additional questions about the result please ask the Genetics team to send an appointment. Please note, patients and their relatives may still be eligible for enhanced mammographic surveillance on account of their family history. Relatives can seek advice about enhanced screening at their local Regional Genetics centre, via a GP referral.

Q: What happens if a mutation is identified?
The Genetics team will inform the patient in writing and will send the patient a copy of the report and the information sheet ‘Receiving a BRCA1 and BRCA2 test result that identifies a mutation’ (MCG IS3) and an appointment for the Genetics clinic. The Cancer team should use the information as appropriate for their cancer management. The Genetics team will discuss with the patient the implications for their future cancer risk and will also evaluate which relatives may be impacted. The processes for cascading the information to relatives will be explained.

Q: What if there is a variant requiring evaluation (VRE) identified?
Very occasionally (<1%), we identify a variant that does not fulfill the criteria for pathogenic mutations, but requires further evaluation. In such cases, an information sheet (MCG IS4) and an appointment with Genetics is sent to the patient. The result and further analyses required are discussed with the patient. Once the additional evaluation has been completed (typically 2-6 months) the patient and clinician are informed of the final management class. Variants are only classified as VREs if there is suggestive evidence of pathogenicity that can potentially be confirmed by additional analyses (e.g. a splicing assay).

Q: What if there is an equivocal result?
No equivocal reports are issued. Genetics / TGL review all the BRCA results, determine the pathogenicity of any identified sequence variants, and interpret their clinical relevance. We have a sophisticated clinical and bioinformatic interpretative system that allows us to do this. The reports thus include clear information about whether or not a pathogenic mutation was identified. Very occasionally (<1%), there are sequence variants that do not fulfill the criteria for pathogenic mutations, but merit further evaluation as described above.

Q: What if new evidence in the future shows a variant is pathogenic?
We keep all variants identified under review and if any are reclassified Genetics will automatically re-issue reports and clear, revised recommendations. It is important to remember that rare sequence variants in these genes are collectively common in the general population (present in about 10%), and the great majority are not pathogenic.

Q: If a mutation is identified who will follow-up the patient’s relatives?
The Genetics team will give the patient a “To whom it may concern letter” to give to relatives. The letter will explain that a cancer predisposition gene mutation has been identified in the family and that relatives can ask their GP to refer them to the Royal Marsden Genetics team or their local genetics service to discuss the implications. This is standard practice in Genetics.

Q: If the patient does not have a BRCA mutation, are there additional genetic tests that should be performed?
Some patients may be eligible for further tests, particularly if they were diagnosed at a particularly young age, if they have multiple primary cancers or if there is an extensive family history of cancer. We recommend that such patients are referred to Genetics.

Q: Who should I contact if I have any questions?
Questions should be addressed to Angela George (Gynae) on angela.george@rmh.nhs.uk or Zoe Kemp (Breast) on zoe.kemp@rmh.nhs.uk.

Please contact cancergenetics@rmh.nhs.uk if you have suggestions for additional questions.

Genetic Unit standard operating procedure for returning results

NO MUTATION

1. Test report, letter (MCG GRL1) + information sheet (MCG IS2) sent to patient[1,2].
2. Test report + letter (MCG GRL1) sent to Cancer Team[1].
3. Genetics appt only sent if requested.

MUTATION

1. Test report, letter (MCG GRL2), information sheet (MCG IS3) + *FHQ sent to patient[1,2].
2. Test report + letter (MCG GRL2) sent to Cancer Team[1].
3. Genetics appt sent to patient.

VARIANT REQUIRING EVALUATION (VRE)

1. Test report, letter (MCG GRL3), information sheet (MCG IS4) + *FHQ sent to patient[1,2].
2. Test report + letter (MCG GRL3) sent to Cancer Team[1].
3. Genetics appt sent to patient.

Notes:

[1] See genetics result letters 1-3 (MCG GRL1-3)

[2] See information sheets 1-3 (MCG IS1-4)

* FHQ = Family history questionnaire

Template genetics results letter for BRCA testing through mainstreaming pathway

MCG GRL1 - NO MUTATION IDENTIFIED

Please insert appropriate text into the grey boxes in the template letter below.

Dear [patient],

You had a BRCA1 and BRCA2 gene test because you have had cancer.

We are writing with the results of the test. The test result is normal. No mutation was identified. We enclose the test report. We also enclose an information sheet which provides further details about this result. We have sent a copy of this letter to your cancer doctor so they know the test was normal.

If you have any questions about this result, please contact the Genetics Unit on [Genetics Unit contact details].

Yours sincerely,
[Genetics Team]

Enclosed**:** Test report, Information sheet MCG IS2
cc**:** [Cancer Team]

Template genetics results letter for BRCA testing through mainstreaming pathway

MCG GRL2 - MUTATION IDENTIFIED

Please insert appropriate text into the grey boxes in the template letter below.

Dear [patient],

You had a BRCA1 and BRCA2 gene test because you have had cancer.

We are writing with the results of this test. The test has identified a mutation (change) in [BRCA1/BRCA2 known as XXXX] in your blood sample. We enclose your test report. This result may have implications for your cancer treatment and/or follow-up. Your cancer doctor will discuss this with you. It also has implications for your future health and may have implications for your relatives.

We enclose an information sheet with some basic information. You may also like to read the information booklet, “A Beginner’s Guide to BRCA1 and BRCA2” which can be downloaded from www.royalmarsden.nhs.uk/brca.

An appointment has been made for you to meet with a member of the Genetics team to discuss the issues in more detail.

The appointment is on [date/time] at [place].

If this appointment is not convenient please contact us on [Genetics Unit contact details] so we can rearrange it.

We enclose a Family History Questionnaire for you to complete and bring to your appointment so we can evaluate which relatives could benefit from gene testing. After your appointment we will provide you with a letter to give to other family members so they can arrange an appointment to discuss testing, should they wish to.

Yours sincerely,
[Genetics Team]

Enclosed: Test report, Information sheet MCG IS3, Family History Questionnaire FHQ
cc**:** [Cancer Team]

Template genetics results letter for BRCA testing through mainstreaming pathway

MCG GRL3 - VARIANT REQUIRING EVALUATION IDENTIFIED

Please insert appropriate text into the grey boxes in the template letter below.

Dear [patient],

You had a BRCA1 and BRCA2 gene test because you have had cancer.

We are writing with the results of this test. The test has identified a [BRCA1/BRCA2] gene variant (change) that requires further evaluation. We enclose the test report. We also enclose an information sheet which provides further details about this result. An appointment has been made for you to meet with a member of the Genetics team to discuss the result in more detail.

The appointment is on [date/time] at [place].

If this appointment is not convenient for you please contact us on [Genetics Unit contact details] so we can rearrange it.

We enclose a Family History Questionnaire for you to complete and bring to your appointment. This information will help us in our evaluations.

Yours sincerely,
[Genetics Team member name]

Enclosed: Test report, Information sheet MCG IS4, Family History Questionnaire FHQ
cc**:** [Cancer Team]
